# The Interaction Between Morphological Awareness and Word Detection Skills in Predicting Speeded Passage Reading in Primary and Secondary School Chinese Readers

**DOI:** 10.3389/fpsyg.2022.802005

**Published:** 2022-03-03

**Authors:** Duo Liu, Zhengye Xu, Li-Chih Wang

**Affiliations:** ^1^Department of Special Education and Counselling, The Education University of Hong Kong, Hong Kong, Hong Kong SAR, China; ^2^Department of Special Education, National Tsing Hua University, Hsinchu, Taiwan

**Keywords:** compensation mechanism, morphological construction, morphemic structure, reading fluency, visual-orthographic processing

## Abstract

Previous studies suggest that morphological awareness (MA) and word detection skills have facilitating roles in reading fluency; however, it is unknown whether they can interplay with each other in such roles. The present study explored the relationships of MA, word detection, and passage reading fluency across ages. In total, 180 Chinese primary and secondary school students, aged from 8.52 to 15.67 years, completed tasks for these aforementioned capacities. After controlling gender, non-verbal intelligence, and reading ability at the word level, the results showed that the participants with higher scores for MA or word detection performed better in passage reading fluency. However, the predictive effect of word detection on reading fluency became weaker as the children became older. The interaction between MA and word detection was positive in younger children, whereas this interaction tended to be negative for older children. The results demonstrated a dynamic interplay between MA and word detection in contributing to passage reading fluency in Chinese children. While it has a positive interaction with word detection on reading fluency in younger children, MA may become a compensator in older children (e.g., over 14 years old) whose word detection skills are less effective in facilitating fluent reading.

## Introduction

Fluent sentence/text reading is an important aspect of reading competency (Nunes et al., 2012) and a critical bridge between word reading and reading comprehension (Kim and Wagner, 2015). Reading fluency has been found to predict a series of school outcomes (Bigozzi et al., 2017). Multiple metalinguistic skills have been shown to contribute to reading fluency, with morphological awareness (MA) one of the most dominant across languages (Kirby et al., 2012; Vaknin-Nusbaum, 2021), and particularly in Chinese (Pan et al., 2016; Zhao et al., 2019). Some other cognitive skills, such as visual-orthographic processing (i.e., the processing of visual information in written scripts), have also been found to be important for children’s reading fluency (e.g., O’Brien et al., 2011; Rakhlin et al., 2019), since natural reading starts from the processing of visual symbols (i.e., letters or Chinese characters). The visual features of the Chinese writing system highlight the importance of visual-orthographic processing in reading in Chinese (Yang et al., 2013). However, no studies so far have investigated how MA and visual-orthographic processing interplay with each other in predicting reading fluency. Furthermore, age could be an interesting factor that may influence their interactions. In the present study, therefore, a word detection task was used to measure Chinese children’s visual-orthographic processing, and its interaction with MA in predicting passage reading fluency across different ages were explored.

Morphological awareness, defined as the ability to reflect on and manipulate the smallest meaningful units and the morphemic structure of words in one’s language (Carlisle, 1995), is important for learning to read in Chinese (e.g., McBride-Chang et al., 2003; Liu et al., 2013). The features of the Chinese language, such as the one-to-one-to-one correspondence among syllable, morpheme, and Chinese character (with only a few exceptions), the dominance of compounding morphology, and the great amount of homophones/homographs, highlight the importance of MA in Chinese children’s literacy development (Liu and McBride-Chang, 2010).

Although few studies have tapped the role of MA in Chinese children’s reading fluency, there is some evidence of their relationship (e.g., Pan et al., 2016; Zhao et al., 2019). Passage reading fluency is the ability to read rapidly with ease, accuracy, appropriate expression, and phrasing (Grabe, 2009). MA, as a critical skill for linking the form (i.e., phonological), semantic, and grammatical information (e.g., Carlisle, 1995; Li and Wu, 2015), can facilitate the quality processing of words in the word-learning process (Perfetti, 2007), and thus help children process the words efficiently and fluently during passage reading.

**FIGURE 1:**
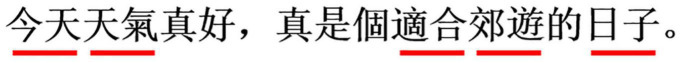
An example of a written Chinese sentence. Words in the sentence were marked with red underlines, which would not be presented in the real reading.

At the same time, since natural reading always begins from the processing of visual symbols (Weiss et al., 2016), many researchers have emphasized the role of visual processes in reading fluency in alphabetic languages (e.g., Bosse and Valdois, 2009; Cunningham et al., 2011). The high visual complexity of Chinese scripts, the arbitrary phonology-to-orthography mapping, and the lack of word boundaries in Chinese scripts (Bai et al., 2008; see Figure 1) reinforce the importance of visual-orthographic processing in fluent reading in Chinese (Siok and Fletcher, 2001; Yang et al., 2013; Liu and Chen, 2020).

Word detection, namely the ability to identify a familiar word accurately and quickly amongst a string of Chinese characters, has been found to be an important predictor of passage reading in Chinese, after word-reading ability is controlled (Liu and Chen, 2020). Word detection is, to some extent, similar to sight word identification in alphabetic languages that has been thought to relate closely to visual-orthographic processing (e.g., Grainger and Hannagan, 2014). In a study by Liu and Chen (2020), word detection acted as a mediator of the association between visual search and passage reading, indicating the embedment of visual processing components in this test. Therefore, in the present study, we expected that word detection should also show importance in predicting passage reading fluency.

Besides their independent roles in predicting reading fluency, MA and word detection may also interplay with each other in implementing their functions. This is an issue that has not been tapped previously. According to the Cognitive Load Theory (Sweller, 2011), one of two cognitive tasks that compete for limited cognitive resources would release some resources for another if the resources required by it are reduced. Efficient visual-orthographic processing, reflected by good performance in word detection, could enhance the processing speed of a given word, and save the limited cognitive resources for further lexical processing, including the morphological aspect, and thus secure the facilitation effect of MA on passage-reading fluency. That is, a positive moderation effect of word detection on the association between MA and passage-reading fluency can be expected.

However, when the developmental issue is considered, the pattern may become less straightforward. Specifically, the importance of visual-orthographic processing to reading has been demonstrated in early Chinese readers, but not older ones, considering that, as a fundamental cognitive ability, it will be developed well when children get older (Ho and Bryant, 1997; Siok and Fletcher, 2001; Yang et al., 2013). At the same time, increases in children’s MA competency in Chinese have been observed across the whole primary school period (Ku and Anderson, 2003), while its association with reading fluency has been found at different grade levels (Li and Wu, 2015). Thus, we assumed that the positive interaction between MA and word detection would be observed in younger readers (e.g., 8–10 years old), because they are under development in both of MA and word detection, and those with better performances in word detection can save more cognitive resources to MA. For older readers (e.g., 13–16 years old), however, along with its development, the predictive role of word detection in reading fluency may decrease, and thus the positive interaction between MA and word detection may disappear. To control the influence of word-reading ability on word detection, Chinese character reading was included as a controller. Non-verbal intelligence was also controlled in the present study.

## Methods

### Participants

In total, 180 typically developing Chinese students (90 males, mean age = 11.76 years, ranging from 8.52 to 15.67) from grade three to grade nine from Taiwan were recruited. There were 39 third graders (21 males, mean age = 8.90 years, ranging from 8.25 to 9.50), 19 fourth graders (8 males, mean age = 9.75 years, ranging from 9.25 to 10.17), 31 fifth graders (12 males, mean age = 11.14 years, ranging from 10.58 to 11.75), 26 sixth graders (15 males, mean age = 11.77 years, ranging from 11.08 to 12.33), 21 seventh graders (10 males, mean age = 13.40 years, ranging from 12.75 to 14.08), 23 eighth graders (14 males, mean age = 14.45 years, ranging from 13.50 to 15.67), and 21 ninth graders (10 males, mean age = 15.25 years, ranging from 14.75 to 15.67). The participants were native speakers of Mandarin Chinese, and had no physical or mental problems with visual, linguistic, or developmental aspects, according to their teachers and parents. Written informed parental consent was obtained for all participants.

### Measures

#### Passage Reading Fluency

A passage reading fluency was adopted from a previous study (Wang, 2020). The passage used in this task contains third-grade-level Chinese characters to reach the function of automaticity in oral reading fluency [for a review, see Fuchs et al. (2001)]. Using these characters can make it easier for the readers to assume that they can process these texts/words accurately and automatically. The passage is a fictional narrative, which is considered as the most reader-friendly type (Marsh and Fazio, 2006). The passage consists of 17 lines, each containing 20 characters, thus an overall total of 340 Chinese characters. The participants were asked to read aloud as many Chinese characters as they could in 1 min, earning one point every time they read a character correctly. The method words-correct-per-minute (WCPM; Hasbrouck and Tindal, 2006), i.e., subtracting the total number of errors from the total number of words read in 1 min, was used to calculate the score for each line. The maximum score of the passage was 340. The Cronbach’s α coefficient was 0.88.

#### Word Detection Task

**FIGURE 2:**
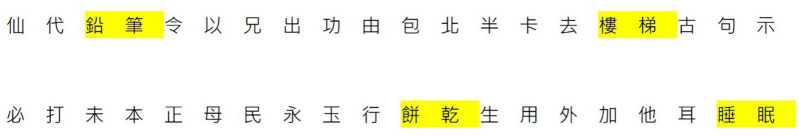
A sample trial in the word detection task. The highlighted items are the targets (i.e., real words).

A word-detection task was adopted from Liu and Chen (2020). The participants were asked to circle two-character words as quickly and accurately as possible in a left-to-right and top-to-bottom direction. There were 16 trials, with two rows in each trial. In each row, two words were embedded in 16 background characters in random positions (see Figure 2). The last character of the first row and the first character of the second row never formed a word. All characters were selected from grade one Chinese textbooks to make sure they would be easy for our participants and were presented in Size 16 BiauKai Font. Because of its nature, this task was designed to capture the visual-orthographic processing skills, rather than knowledge about the concepts of words (Lin et al., 2011) or word reading accuracy, as evident in the study by Liu and Chen (2020). A word-detection efficiency index was calculated using a formula: efficiency = (number of correctly crossed out words−number of errors)/time (in second); the number of errors included the number of target words that were missed and the number of non-words that were crossed out incorrectly. “Time” was the total time for a child to complete the task. The Cronbach’s α coefficient was 0.89.

#### Morphological Awareness

A morphological construction task (Liu and McBride-Chang, 2010) was adopted to measure MA. The participants were presented orally with one two/three-character compounded word with given scenarios. For example, “if we see the sun rising in the morning, we call that/ri4 sheng1/(sunrise).” Then, the participants were asked to construct a novel word to correspond to a similar scenario, for example, “What should we call the phenomenon of the moon rising?” (Answer:/yue4 sheng1/(moonrise). There were 15 questions; one point was assigned for each correct answer. The maximum score was 15. The overall Cronbach’s α coefficient was 0.63.

#### Chinese Character Reading

A standardized test, the Chinese Character Recognition Test (Huang, 2001), was used to examine the participant’s reading ability at the word level. The participants were required to read 200 characters aloud one-by-one. These characters were sorted by increasing difficulty. Testing stopped when the child failed to recognize 20 consecutive characters. One point was awarded for each correct character; the maximum score was 200. The Cronbach’s α coefficient was 0.94.

#### Raven’s Standard Progressive Matrices–Parallel

This is a standardized test of non-verbal intelligence (Chen and Chen, 2006) that consists of 60 items with increasing difficulty. For each item, the children were asked to identify the missing element from six or eight options, according to a pattern. The number of correctly responded items was the raw score for this test. Based on the Taiwanese norm established by the Chinese Behavioral Science Co., in 2006, the raw score of each participant was converted to the standard score in terms of the participant’s age. Based on our sample, the Cronbach’s α coefficient was 0.81.

## Results

### Preliminary Analysis

The means and standard deviations for age and the measures, as well as the correlations between them, are summarized in Table 1. The missing data (age from one participant and word-detection score from another) were replaced with corresponding means by grade. An inspection of skew and kurtosis statistics suggested that the data were normally distributed. The correlation analysis showed that age, as well as non-verbal intelligence, was positively correlated with word detection, MA, reading fluency, and Chinese character reading. Moreover, word detection, MA, reading fluency, and Chinese character reading were significantly correlated with each other.

**TABLE 1:** Means, SDs, and correlations among measures.

	Maximum score	*M* (SD)	Range	Skewness	Kurtosis	1	2	3	4	5	6
1 Age	/	11.76 (2.25)	8.52–15.67	0.18	–1.23	–					
2 Non-verbal intelligence	/	99.66 (15.70)	56–128	–0.21	–0.45	0.07	–				
3 Word detection	/	0.26 (0.12)	0.03–0.84	1.32	3.3	0.61***	0.25**	–			
4 Morphological awareness	15	13.56 (1.68)	6–15	–1.98	4.93	0.29***	0.23**	0.30***	–		
5 Reading fluency	340	192.24 (40.03)	78–228	–1.06	0.19	0.57***	0.24**	0.48***	0.50***	–	
6 Chinese character reading	200	128.91 (32.19)	33–189	–0.54	–0.12	0.71***	0.27***	0.62***	0.41***	0.76***	–

*N = 180.*

**p < 0.01; ***p < 0.001.

*/Not applicable.*

### Linear Mixed Model Analysis

A linear mixed model (LMM), in which the fixed effects of interest and random effects were taken into account simultaneously, was constructed by using the lme4 package in R 3.4.2 (R Core Team, 2017) for analyzing reading fluency. The dependent variable was the score of each line in the passage reading fluency task. Word detection, MA, age, and their interactions were entered as fixed variables. In addition, the fixed variables included covariates, i.e., gender (the contrast between girls and boys, the boys is the reference), non-verbal intelligence, and Chinese character reading. The random factors included random intercepts by participants and items. In addition, random slopes of word detection, MA, and age by items were involved. The continuous factors (i.e., reading fluency, word detection, MA, age, non-verbal intelligence, and Chinese character reading) were centered by a *z*-score transformation of raw scores to overcome the problem of collinearity.

**FIGURE 3:**
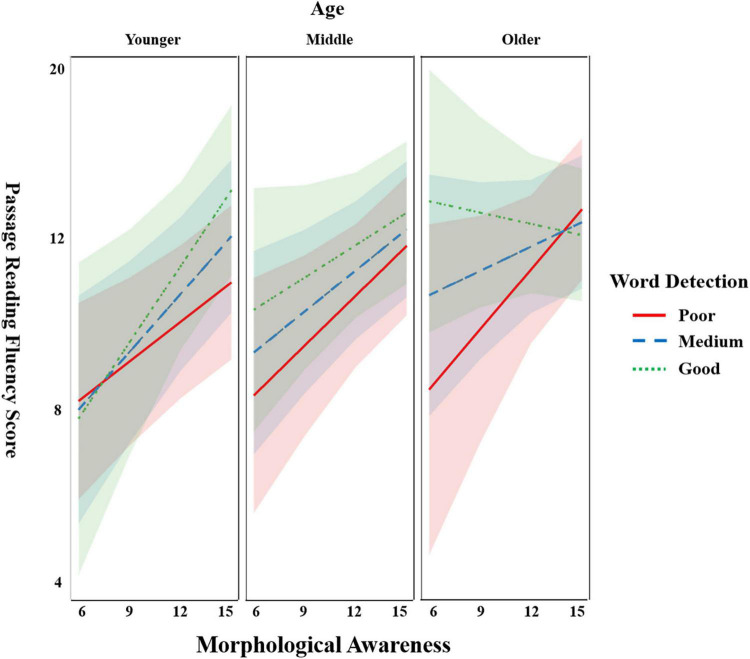
The interaction effect of word detection, morphological awareness (MA), and age on reading fluency. Figure 3 was developed based on the results of a linear mixed model (LMM), in which age and word detection were entered as continuous factors. To display the results visually, three representative ranges of age (i.e., Younger, Middle, and Older) and of word detection (i.e., Poor, Medium, and Good) were selected according to their corresponding Mean (age: 11.76; word detection: 0.26) and *SD* (age: 2.25; word detection: 0.12). Younger age/Poor word detection = smaller than (Mean–1*SD*); Middle age/Medium word detection = between (Mean–1*SD*) and (Mean + 1*SD*); and Older age/Good word detection = larger than (Mean + 1*SD*).

The results (see Table 2) showed that the effects of word detection (ß = 0.07; *p* = 0.04) and MA (ß = 0.12; *p* = 0.001) were significant. The score for reading fluency increased with the score for word detection, as well as with the score for MA. The effect of Chinese character reading (ß = 0.25; *p* < 0.001) was significant. The effects of age, gender, and non-verbal intelligence were not significant (*p*s > 0.05). The children with higher scores on Chinese character reading had higher scores on reading fluency. The interaction of word detection and age on reading fluency was significant (ß = −0.07; *p* = 0.003). The facilitating effect of word detection on reading fluency was larger in younger children than in older ones. The interaction between word detection and MA (ß = −0.01; *p* = 0.81) and the interaction between MA and age (ß = −0.02; *p* = 0.37) were not significant. More importantly, the three-way interaction (i.e., word detection × age × MA) was significant (ß = −0.06; *p* = 0.01). The interaction effect of word detection and age was impacted by MA. As shown in Figure 3, MA and word detection tended to interact positively with each other for younger children, while this interaction became negative for older children.

**TABLE 2:** Results of the linear mixed model for reading fluency.

Variables	ß	*SE*	*t*	*P*
WD	0.07	0.03	2.05	0.04
MA	0.12	0.04	3.31	0.001
Age	0.03	0.04	0.75	0.45
WD × MA	–0.01	0.03	–0.25	0.81
WD × Age	–0.07	0.02	–2.94	0.003
MA × Age	–0.02	0.03	–0.89	0.37
WD × Age × MA	–0.06	0.02	–2.81	0.01
**Controls**				
Gender	0.08	0.05	1.45	0.15
Non-verbal intelligence	0.02	0.03	0.90	0.37
CCR	0.25	0.06	3.95	<0.001

*Gender in the model presents the contrast between boys and girls, with the boy as the reference.*

*WD, word detection; MA, morphological awareness; CCR, Chinese character reading.*

## Discussion

This study is among the first to examine the relationships among word detection, MA, and passage-reading fluency across multiple age groups in Chinese children. Besides the main effects of word detection and MA, we also found that the prediction of MA on passage reading fluency remained stable for children aged from 8.52 to 15.67, while the effect of word detection became weaker for the older children. More interestingly, we found a significant three-way interaction among age, word detection, and MA, which will be discussed in detail later.

The significant predictive effect of MA on passage reading fluency found in the present study and its constant importance across ages were consistent with previous studies (Li and Wu, 2015; Pan et al., 2016; Zhao et al., 2019). MA is thought to be a synthesized skill that covers the processing of form, semantic and grammatical information (e.g., Carlisle, 1995; Li and Wu, 2015), and, thus, is critical for establishing the quality processing of words (Perfetti, 2007), which is related closely to fluent reading (Manolitsis et al., 2017; Desrochers et al., 2018). On top of the existing literature on the association between MA and reading fluency in preschool and primary school Chinese children (Li and Wu, 2015; Pan et al., 2016; Zhao et al., 2019), the present study expanded the age range to secondary school.

The role of word-detection skills in reading fluency was consistent with previous findings on the association between visual-orthographic processing and reading fluency in Chinese (Siok and Fletcher, 2001; Luo et al., 2013), as was its negative interaction with age (Ho and Bryant, 1997; Yang et al., 2013). After controlling for character-reading ability, word detection was thought to mainly reflect the processing of visual-orthographic information during the efficient identification of words that the children were familiar with. As we mentioned earlier, a serial of features of the Chinese writing system highlights the importance of visual-orthographic processing in reading. At the same time, as a basic cognitive ability, its predictive role in reading in Chinese is limited to younger children (Yang et al., 2013).

Nevertheless, when the interaction among MA, word detection, and age was considered in the model, a complex, but at the same time interesting, picture was demonstrated. As we expected, MA and word detection showed a positive interaction in younger children, indicating that the competency in either of the two skills could help save cognitive resources for the other in contributing to facilitate fluent reading. This finding fits the Cognitive Load Theory (Sweller, 2011) on the completion of limited cognitive resources of two cognitive tasks. In older children, on the other hand, the interaction between MA and word detection became negative. First, this finding supports our expectation that the positive interaction between the two skills would disappear in older children. Second, and beyond that, the negative interaction in older children indicated that MA showed a stronger predictive effect on children whose word-detection skills were relatively poorer. In other words, MA may become a compensator in children whose word-detection skills were less effective in facilitating fluent reading.

Although rare in previous research, the compensation mechanism was found by Heim et al. (2010) in their study of the interaction between phonological and visual processing. These researchers found that children with dyslexia who were poor in phonological processing would place higher demands on visual attention. The present study provided evidence of the interaction of another two important factors for reading, i.e., MA and word detection, and emphasized the dynamic nature of such interaction across ages in typically developing children.

There were some limitations in the present study. First, since it was a small-scale study, we did not obtain a large sample in each grade, which may have affected the power of the analyses. Second, a single MA task was used across all grades. The suitability of this measure may vary for children at different grade levels. This may be the reason why we got a relatively low reliability for this measure. In the future, larger-scale studies on this topic could be conducted, so that more relevant measures in each domain (e.g., visual-orthographic processing and metalinguistic awareness) can be administered.

Despite these limitations, the present study is among the first to explore the dynamic interactions between MA and word detection, with age taken into consideration. It is hoped that this study can provide a novel direction for researchers to further explore the possible relationships of factors at different levels and different models, so that a more comprehensive picture can be drawn for understanding the development of literacy.

## Data Availability Statement

The raw data supporting the conclusions of this article will be made available by the authors, without undue reservation.

## Ethics Statement

The studies involving human participants were reviewed and approved by the Human Research Ethics Committee at The Education University of Hong Kong. Written informed consent to participate in this study was provided by the participants’ legal guardian/next of kin.

## Author Contributions

DL, ZX, and L-CW contributed to the conception and design of the study and wrote sections of the manuscript. DL wrote the first draft of the manuscript. All authors contributed to the statistical analysis, manuscript revision, read, and approved the submitted version.

## Conflict of Interest

The authors declare that the research was conducted in the absence of any commercial or financial relationships that could be construed as a potential conflict of interest.

## Publisher’s Note

All claims expressed in this article are solely those of the authors and do not necessarily represent those of their affiliated organizations, or those of the publisher, the editors and the reviewers. Any product that may be evaluated in this article, or claim that may be made by its manufacturer, is not guaranteed or endorsed by the publisher.
